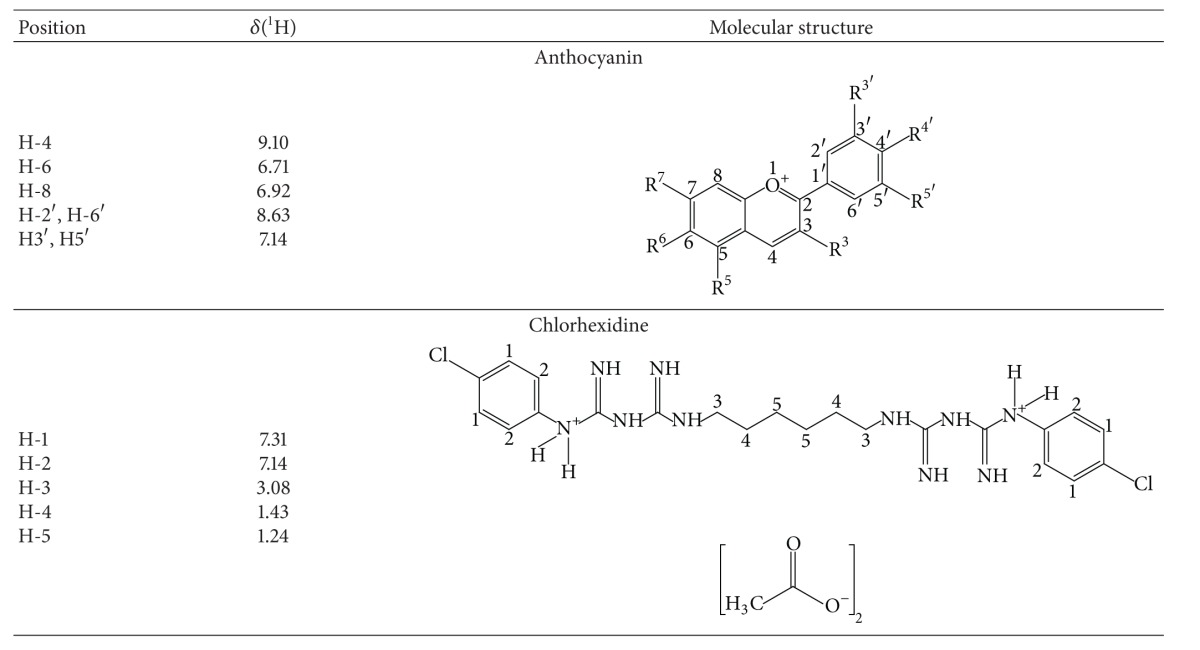# Anthocyanin Incorporated Dental Copolymer: Bacterial Growth Inhibition, Mechanical Properties, and Compound Release Rates and Stability by ^1^H NMR

**DOI:** 10.1155/2014/289401

**Published:** 2014-02-18

**Authors:** Halyna Hrynash, Vinay Kumar Pilly, Alexandra Mankovskaia, Yaoyang Xiong, Getulio Nogueira Filho, Eduardo Bresciani, Céline Marie Lévesque, Anuradha Prakki

**Affiliations:** ^1^Department of Clinical Sciences-Restorative, Faculty of Dentistry, University of Toronto, 124 Edward Street, Room 352C, Toronto, ON, Canada M5G 1G6; ^2^Department of Biological and Diagnostic Sciences-Dental Public Health, Faculty of Dentistry, University of Toronto, 124 Edward Street, Toronto, ON, Canada M5G 1G6; ^3^Department of Biological and Diagnostic Sciences-Oral Microbiology, Faculty of Dentistry, University of Toronto, 124 Edward Street, Toronto, ON, Canada M5G 1G6; ^4^Department of Prosthodontics, School of Medicine, Shanghai Jiaotong University, 639 Zhizaoju Road, Shanghai 9th Hospital, Shanghai 200011, China; ^5^Department of Biological and Diagnostic Sciences-Preventive Dentistry, Faculty of Dentistry, University of Toronto, 124 Edward Street, Toronto, ON, Canada M5G 1G6; ^6^Department of Restorative Dentistry, Institute of Science and Technology, Universidade Estadual Paulista (UNESP), Avenida Eng. Francisco José Longo 777, 12245-000 São José dos Campos, SP, Brazil

## Abstract

*Objective.* To evaluate bacterial growth inhibition, mechanical properties, and compound release rate and stability of copolymers incorporated with anthocyanin (ACY; *Vaccinium macrocarpon*). *Methods.* Resin samples were prepared (Bis-GMA/TEGDMA at 70/30 mol%) and incorporated with 2 w/w% of either ACY or chlorhexidine (CHX), except for the control group. Samples were individually immersed in a bacterial culture (*Streptococcus mutans*) for 24 h. Cell viability (*n* = 3) was assessed by counting the number of colony forming units on replica agar plates. Flexural strength (FS) and elastic modulus (*E*) were tested on a universal testing machine (*n* = 8). Compound release and chemical stability were evaluated by UV spectrophotometry and ^1^H NMR (*n* = 3). Data were analyzed by one-way ANOVA and Tukey's test (**α** = 0.05). *Results.* Both compounds inhibited S. mutans growth, with CHX being most effective (*P* < 0.05). Control resin had the lowest FS and E values, followed by ACY and CHX, with statistical difference between control and CHX groups for both mechanical properties (*P* < 0.05). The 24 h compound release rates were ACY: 1.33 *μ*g/mL and CHX: 1.92 *μ*g/mL. ^1^H NMR spectra suggests that both compounds remained stable after being released in water. *Conclusion.* The present findings indicate that anthocyanins might be used as a natural antibacterial agent in resin based materials.

## 1. Introduction

Caries is an endogenous infection that causes lesions in enamel, cementum, and dentin by the action of carbohydrate-rich diet associated with bacteria able to produce, by glycosyltransferase enzyme, insoluble bioadhesive polysaccharides [1]. It mediates formation of plaque and accumulation of mutans group streptococci, allowing them to adhere to dental structure. These bacteria produce organic acids (lactic, acetic, propionic, and formic) that lead to loss of tooth mineral initiating the process of cavitation. The synthetic antiseptic chlorhexidine (CHX) has been used as mouth rinse and has also been incorporated to some dental materials for the prevention of caries and caries progression. This compound, however, may present disadvantages if in routine use such as bacteria resistance [2], cytotoxicity, and change in taste perception [3, 4]. As a consequence, research has been dynamic in trying to find alternative, natural-safe antibacterial compounds to be used in mouthwashes and novel biomaterials [5], such as different resin formulations that could act on cariogenic sites.

In recent studies, some benefits related to polyphenols in cranberry (*Vaccinium macrocarpon*) juice or extracted from cranberry fruit have been established. It has been demonstrated that cranberry polyphenols have the ability to decrease the cell surface hydrophobicity of streptococcal bacteria (*S. sobrinus* and *S. mutans*) [6] and adhesion between cariogenic bacteria and enamel-like structures (hydroxyapatite beads) [7] and enamel-like structures pretreated with glucans [8]. Other studies confirmed that cranberry extracts are not only able to inhibit the adhesion of *S. sobrinus* to enamel-like structures coated with saliva [9], but also led to desorption of the same bacterial species from an artificial dental biofilm [10]. More recently, the ability of cranberry polyphenols in reducing the formation of biofilm by *S. mutans in vitro* and dental caries development *in vivo* (Sprague-Dawley rats) has also been reported [11]. Moreover, Bodet and colleagues [12] reported that low concentrations of cranberry extract inhibited the secretion of MMP-3 and MMP-9 by the gingival fibroblasts and macrophages following stimulation by the LPS of *Aggregatibacter actinomycetemcomitans*, a causative agent involved in periodontal disease. Their results also showed that the cranberry extract inhibited the catalytic activity of both enzymes and elastase. As dentinal proteases are believed to play an important role in the progression of carious lesions, inhibition of MMPs such as MMP-9 might also help preventing caries progression [13].

Most studies that evaluated the effects of cranberry polyphenols on cariogenic bacteria and MMP activity used a fraction of cranberries called the nondialyzable material (NDM), which is obtained by dialysis of concentrated cranberry juice [6, 7, 9, 10, 12]. The content of various cranberry polyphenols (phenolic acids, anthocyanins, flavonols, flavan-3-ols, and proanthocyanidins) in cranberry NDM is subjected to variations depending on seasonal and varietal effects. Although, the anthocyanins, flavonols, and proanthocyanidins are among the most abundant classes of polyphenols and have been associated with the health promoting benefits of cranberry [14], the biological action and stability after incorporation into resins and therefore potential usefulness of these compounds are still unclear. In view of the forgoing discussion, the objective of this study was to evaluate bacteria growth inhibition (against *S. mutans*), mechanical properties, and compound stability of a dental resin incorporated with an ACY-rich cranberry extract. Resins containing CHX will be also evaluated as a control.

## 2. Material and Methods

### 2.1. Bacterial Strain, Growth Condition, and Chemicals

*S. mutans* UA159 strain isolated from a child with active caries was used in this study [15]. *S. mutans* was cultivated in Brain Heart Infusion (BHI) broth at 37°C in air with 5% CO_2_. BHI broth agar plates were prepared using 1.5% (w/v) agar. Bis-GMA, TEGDMA, the photosensitizer camphorquinone (CQ), reducing agent 2-(dimethylamino)ethyl methacrylate (DMAEMA), cranberry ACY (25%, Changsha Nutramax Inc., Changsha, China), and CHX diacetate salt hydrate (Sigma-Aldrich, St. Louis, MO, USA) were all used as received.

### 2.2. Formulation of Comonomers

The experimental resin formulation was prepared combining Bis-GMA at 70 mol% and TEGDMA at 30 mol%. Except for the control group (no compounds added), each formulation was randomly mixed with either ACY or CHX at 2 w/w% comprising a total of 3 groups. Comonomers were activated for visible light polymerization by the addition of CQ and DMAEMA (0.2 w/w% each). For all tests (*n* = 3) except flexural properties (*n* = 10), specimens were fabricated inside an acrylic matrix with internal dimensions of 5 mm diameter × 3 mm height. For flexural properties, specimen dimensions were 2 mm width × 2 mm height × 25 mm length. Unpolymerized material was sandwiched between two polyester strips over a glass-mixing tablet. Polymerization was done on both sides by a visible light curing unit (Demi LED, Kerr Co., WI, USA) for 40 s delivering uninterrupted 540 mW/cm^2^, verified by a radiometer (Model 100, Demetron Research Co., CT, USA). A UV-Vis Spectrophotometer (Synergy HT BioTEK, Winooski, VT, USA) was used to evaluate the compound release rates of all groups in triplicate after 24 h water storage (maximum absorption peaks were ACY: 522 nm and CHX diacetate: 257 nm).

### 2.3. Bacterial Viability Assay

Overnight cultures of *S. mutans* UA159 were diluted (1 : 20) into 1.0 mL of fresh BHI broth followed by addition of one resin sample per test tube. The reaction mixtures were incubated at 37°C for 24 h under constant agitation. The next day, the cells were sonicated and aliquots of 20 *μ*L of each test tube plated on BHI agar for CFU determination. The colonies were counted after 48 h of incubation. The percentage of cell survival corresponded to the number of viable cells after treatment divided by the total number of viable cells in the untreated sample.

### 2.4. Flexural Strength (FS) and Modulus of Elasticity (E)

After polymerization, specimens were finished and individually stored in deionized water at 37°C for 24 h prior to testing. The flexural properties were evaluated by a three-point bending test (Instron Universal Testing Machine, Canton, MA, USA) at a cross-head speed of 0.5 mm/min. Flexural strength was obtained by measuring the load at fracture, and the elastic modulus was calculated from the recorded load-deflection curves [16].

### 2.5. Stability of Compound Incorporated into Copolymer Using Proton NMR (Nuclear Magnetic Resonance)

Single-resonance, ^1^H NMR spectra were collected for the compounds alone and extracted from resin in deionized water (after 24 h), followed by lyophilization, from the loaded copolymers for analysis of compound stability [17]. The spectrometer (Nyago-vnmrs500, Agilent Technologies, ON, Canada) was operated at 500 MHz proton. The temperature required for a 90° pulse was 25°C. The solvent used was D_2_O. In these spectra, signal intensity is plotted versus the chemical shift, measured in ppm.

### 2.6. Statistical Analyses

Data for bacterial viability assay, flexural strength, and elastic modulus were evaluated by one-way ANOVA followed by Tukey's post hoc test at *α* = 0.05.

## 3. Results and Discussion

### 3.1. Results

**Figure 1:**
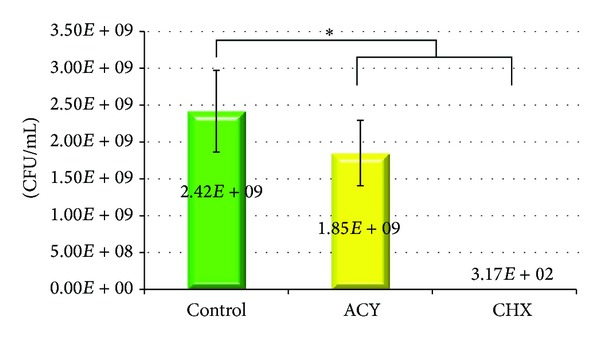
Survival of *S. mutans* (UA159) in response to treatment with Bis-GMA/TEGDMA-based resins containing ACY or CHX (CFU: colony forming units). The vertical bars denote average standard deviation; *denotes statistical significance between groups (*P* < 0.05).

The 24 h mean drug release rates in deionized water were 1.33 ± 0.0 *μ*g/mL for ACY incorporated resin and 1.92 ± 0.4 *μ*g/mL for CHX incorporated resin. The bacteria viability assay for ACY or CHX containing resins is represented in Figure 1. There was a statistical difference among all groups in the percentage bacteria survival rates. The control resin had 100% survival, followed by ACY-resin with 74.6% survival and by CHX-resin which showed virtually no bacteria survival (*P* < 0.05). Mean FS and *E* values are shown in Table 1. The control resin had the lowest FS and *E* values, followed by ACY and by CHX. The values between control and CHX groups were statistically different for both properties (*P* < 0.05).

**Figure 2:**
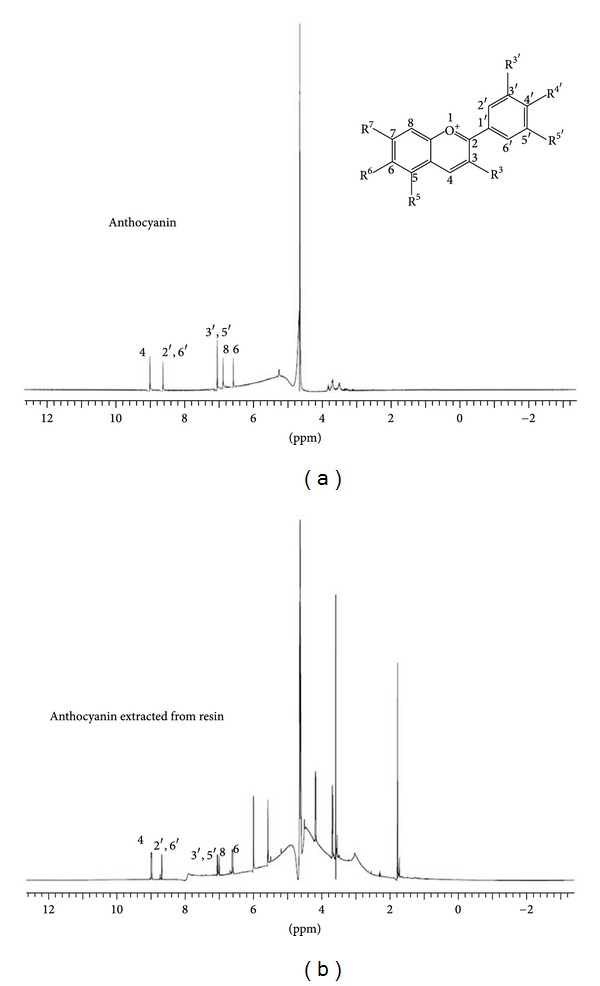
^1^H NMR spectra of (a) anthocyanin compound; (b) anthocyanin released by dental copolymer.

After the assessment of ^1^H NMR spectra, the signals for ACY and CHX (Table 2) have been detected at the resin extracted medium. Representative spectra for ACY pure compound and extracted from resin are depicted as Figures 2(a) and 2(b).

### 3.2. Discussion

Anthocyanins are plant extracts widely found in many berries, dark grapes, cabbages, and other pigmented plants. Chemically, they belong to a widespread class of phenolic compounds collectively named as flavonoids. The differences between individual anthocyanins are related to the number of hydroxyl groups in the molecular structure as well as the nature and number of sugars and the position of these pendent molecules [18, 19]. Many health benefits have been related to the consumption of cranberry extracts (i.e., anthocyanins). An example is the prevention of urinary tract infections due to bacterial antiadhesion activity against both antibiotic susceptible and resistant strains of uropathogenic P-fimbriated *Escherichia coli *bacteria [20, 21] and, also, prevention of gastric cancer (by inhibiting adhesion of *Helicobacter pylori*) [22] as well as prevention of oral diseases like dental caries and periodontitis [23].

The present results support previous findings on antibacterial property of cranberry extracts [6, 10, 11]. The ACY group was able to inhibit growth of *S. mutans*, although with far less effectiveness when compared to CHX. Differences in results are likely related to several factors, for instance, the purity of compound, as the ACY used in this study is only 25% pure (this was the maximum purity that could be commercially obtained for the cranberry anthocyanin) as compared to 100% purity of CHX. The amount of drug released is another important aspect to be taken into consideration as higher values were obtained for CHX. Additionally, previous results [24] have shown that incorporation of CHX at 2% ratio into Bis-GMA/TEGDMA resin leads to an amount of released compound in similar ranges to MIC (minimum inhibitory concentration) value for CHX in 1 mL of water (MIC values for the evaluated ACY compound and CHX in BHI broth are 500 *μ*g/mL and 2 *μ*g/mL, resp.; nonpublished data), which may explain the pronounced *S. mutans* growth inhibition.

The incorporation of both compounds had apparently a reinforcing effect on resins leading to higher flexural strength and modulus property. The ACY group values did not, however, statistically differ from any of the remaining groups. A decrease in resin conversion has very likely played a role in the mean values of ACY mechanical properties, as the cranberry polyphenols are naturally pigmented in red color which affects translucency and polymerization light propagation through the resin sample [25].

In this study, the stability of released compounds into aqueous media was chosen to be analyzed by ^1^H NMR [17]. In these spectra, the chemically different protons (^1^H nuclei) in the sample resonate at different frequencies because they are shielded more or less by the electrons that surround them. The NMR spectrum is treated like a fingerprint of the chemical structure of the molecule. The strong line at *∼*4.65 ppm (Figures 2(a) and 2(b) and seen in all obtained spectra) arises from the small amount of residual water in the solvent. For both ACY and CHX, all expected signals were observed in the leached media (Table 1) as compared to the NMR of the sole compounds, which suggests stability of the released compounds. For ACY spectra, the congested signals between 3.58 ppm and 4.05 ppm correspond to the sugar region [26]. Other signals observed in Figure 2(b) might correspond to nonreacted monomers and impurities released by the comonomer.

## 4. Conclusions

The present findings indicate that anthocyanins might be used as a natural antibacterial agent in resin based materials.

## Figures and Tables

**Table 1:** Mean flexural strength (FS) and modulus of elasticity (*E*) of experimental resins.

Groups	FS (MPa) ± s.d.	*E* (GPa) ± s.d.
Control	66.2 ± 8.8^a^	1.7 ± 0.3^a^
ACY	94.2 ± 26.0^a,b^	2.2 ± 0.7^a,b^
CHX	103.6 ± 12.0^b^	3.1 ± 0.7^b^

Same lower case letters indicate no statistical difference within each column.

**Table 2:** ^
1^H NMR spectroscopic data of the anthocyanin and chlorhexidine diacetate released from a dental copolymer.